# 1-(Triethoxysilyl)buta-1,3-dienes—New Building Blocks for Stereoselective Synthesis of Unsymmetrical (*E*,*E*)-1,4-Disubstituted 1,3-dienes

**DOI:** 10.3390/ma8115378

**Published:** 2015-10-28

**Authors:** Justyna Szudkowska-Frątczak, Mariusz Taczała, Piotr Pawluć

**Affiliations:** 1 Faculty of Chemistry, Adam Mickiewicz University, Umultowska 89b, Poznan 61-614, Poland; 2 Center for Advanced Technologies, Adam Mickiewicz University, Umultowska 89c, Poznan 61-614, Poland; j.sz@amu.edu.pl (J.S.-F.); mariusztaczala@gmail.com (M.T.)

**Keywords:** buta-1,3-dienes, organosilicon dienes, C–C bond formation, Hiyama cross-coupling, palladium catalyst

## Abstract

A convenient methodology for the highly stereoselective synthesis of unsymmetrical (1*E*,3*E*)-1,4-disubstituted 1,3-dienes based on palladium-catalyzed Hiyama cross-coupling reaction of 1-(triethoxysilyl)-substituted buta-1,3-dienes with aryl iodides is reported.

## 1. Introduction

Highly conjugated π-electron compounds such as aryl-substituted dienes and polyenes have gained a lot of attention because of the wide range of their applications in functional materials such as organic fluorescent probes, electroluminescent devices, and nonlinear optical materials [1,2,3,4,5].

Among the syntheses developed to access aryl-substituted buta-1,3-dienes, the transition metal-catalyzed cross-coupling reactions are of prime importance because of their high stereoselectivity [6]. A number of methodologies for the stereoselective preparation of 1,4-disubstituted buta-1,3-dienes based on the palladium-catalyzed cross-coupling of vinyl halides with alkenyl-substituted organometallic compounds of boron [7], tin [8], zinc [9], silicon [10] or zirconium [11,12] have been developed over the last three decades. The complementary synthetic routes involving 1,4-bis-metallated 1,3-butadienyl building blocks are represented by the palladium-catalyzedcross-coupling of aryl or alkenyl halides with 1,4-bis(silyl)- [13,14], 1,4-bis(stannyl)- [15,16], 1,4-bis(boryl)- [17] or 1-boryl-4-stannylbuta-1,3-dienes [18,19,20]. An alternative approach based on cross-coupling reactions of organometallic reagents with 1,4-diiodobuta-1,3-diene has also been reported [21,22].

The palladium-catalyzed and fluoride-promoted cross-coupling of unsaturated organosilicon compounds with aryl or alkenyl halides (Hiyama coupling) has been recently employed as a mild and efficient alternative to the well-established Stille, Negishi, and Suzuki reactions, taking intoaccount the commercial availability, high stability, and low toxicity of silicon derivatives [23,24,25]. In view of the above advantages, we have successfully applied various unsaturated organosilicon precursors such as (*E*)-silylstyrenes [26,27,28], 1,1-bis(silyl)alkenes [29,30], (*E*)-1,2-bis(silyl)alkenes [31] and vinylcyclosiloxanes [32] as versatile double-bond equivalents in the construction of π-conjugated systems.

On the other hand, reports on the successful cross-coupling of silylated buta-1,3-dienes with aryl or alkenyl halides are strongly limited, mainly due to the complexity of their synthesis. Denmark has reported an efficient [Pd_2_(dba)_3_]-catalyzed coupling of 1,4-bis(silyl)buta-1,3-dienes containing two distinct silyl groups (-SiMe_2_OH and -SiMe_2_Bn) with aryl iodides to construct unsymmetrical 1,4-diaryl-1,3-butadiene derivatives [13]. The synthesis was possible thanks to the difference in reactivity between the silanol and silyl groups and the application of conditions developed by Denmark and co-workers for the efficient coupling of silanols with aryl halides. The starting 1,4-bis(silyl)buta-1,3-dienes were obtained by a sequential three-step procedure: rhodium-catalyzed ethynylsilane dimerization, deprotection of the terminal alkyne, and platinum-catalyzed hydrosilylation. This method has been successfully extended to the cross-coupling with alkenyl iodides and applied as a key step in the synthesis of immunosuppressive agent RK-397 [33].

**Scheme 1:**
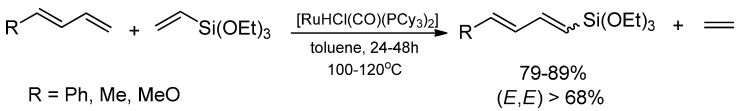
Synthesis of 1-(triethoxysilyl)buta-1,3-dienes.

Recently, we have reported a new method for the synthesis of 1-silyl-substituted buta-1,3-dienes, based on the [RuHCl(CO)(PCy_3_)_2_]-catalyzed silylative coupling of terminal (*E*)-1,3-dienes with vinylsilanes [34]. The reaction provides a facile and straightforward access to (*E*,*E*)-dienylsilanes, including alkoxy-substituted silanes in a highly stereoselective fashion (Scheme 1).

Since the starting 1-(triethoxysilyl)buta-1,3-dienes can be easily prepared with good yield in a one-step process from inexpensive and commercially available substrates, we have envisaged that they could be used as coupling partners for the stereoselective synthesis of 1,4-disubstituted buta-1,3-dienes via Hiyama coupling with aryl or alkenyl iodides. Therefore, herein we report our results on the use of 1-(triethoxysilyl)buta-1,3-dienes as new platforms for the installation of aryl groups onto the C=C core which leads to unsymmetrically (*E*,*E*)-1-aryl- or (*E*,*E*)-1,4-diaryl-substituted buta-1,3-diene derivatives.

## 2. Results and Discussion

Having established an efficient protocol for the highly selective synthesis of 1-silyl-buta-1,3-dienes, we subsequently investigated their reactivity towards selected aryl and alkenyl iodides under Hiyama cross-coupling conditions. Initial studies were carried out using 1-phenyl-4-(triethoxysilyl)buta-1,3-diene (mixture of isomers: (*E*,*E*)/(*E*,*Z*)/(*Z*,*Z*) = 83:15:2) and iodobenzene in the presence of [Pd_2_(dba)_3_] catalyst (4 mol % Pd) and tetrabutylammonium fluoride (TBAF) (2 equiv.) as an activator. After several attempts, we found that its reaction with 1.2 equiv. of aryl iodide conducted in tetrahydrofuran (THF) at 65 °C for 24 h exclusively afforded the coupling product (*E*,*E*)-1,4-diphenylbuta-1,3-diene 1, as a single stereoisomer in 89% yield (Table 1, entry 1). Similar reactivity of 1-phenyl-4-(triethoxysilyl)buta-1,3-diene was observed with other aryl iodides containing electron-withdrawing or electron-donating groups (Table 1, entry 2–4). The stereoselectivity of the Hiyama coupling was high. Although the starting silyldiene consisted of a mixture of geometrical isomers, in all cases the (*E*,*E*) double-bond geometry was strongly favored (99%) as measured by ^1^H NMR (Nuclear Magnetic Resonance Spectroscopy) and GC-MS. (Gas chromatography–mass spectrometry). The (*E*,*E*)-1,4-diarylbuta-1,3-dienes 1–4 were isolated and characterized spectroscopically (see Supporting Information; Figures S1–S8). Although the stereochemistry of dienes 1–4 cannot be directly derived from the ^1^H NMR spectra on the basis of the protons of the diene moiety, the analysis of spin systems by means of MestReC NMR software (Mestrelab Research, Santiago de Compostela, Spain) [21] as well as comparison with literature data [35,36,37,38] allowed us to confirm the diene structure.

**Scheme 2:**
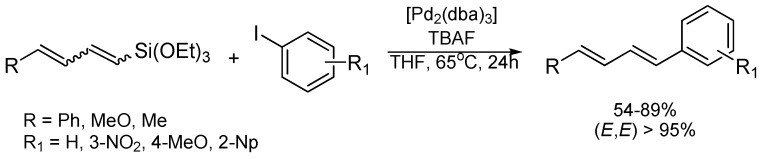
Synthesis of (*E*,*E*)-1,4-disubstituted buta-1,3-dienes.

The palladium-catalyzed Hiyama coupling proceeded efficiently also for other 1-(triethoxysilyl)-substituted dienes such as 1-methoxy-4-(triethoxysilyl)buta-1,3-diene (mixture of isomers: (*E*,*E*)/(*E*,*Z*)/(*Z*,*Z*) = 68:20:12) (Table 1, entry 5–6) or 1-(triethoxysilyl)penta-1,3-diene (mixture of isomers: (*E*,*E*)/(*E*,*Z*)/(*Z*,*Z*) = 71:16:13) (Table 1, entry 7). The noteworthy feature of these processes is that the formation of 1-substituted buta-1,3-diene (via protodesilylation) was suppressed. The formation of biaryls (by competitive *homo*-coupling of aryl iodides) was observed under given conditions in 5%–10% yield. It is worth noting that the Hiyama coupling processes proceeded in a highly stereoselective manner to yield products containing (*E*,*E*)-dienes as predominant compounds; however, trace amounts of the respective (*E*,*Z*) isomers (1%–5%) were also detected using the GC-MS method (Scheme 2). The (*E*,*E*)-1-aryl-4-methoxybuta-1,3-dienes 5–6 and (*E*,*E*)-1-arylpenta-1,3-diene 7 were isolated and characterized spectroscopically (see Supporting Information; Figures S9–S14).

**Table 1:** Hiyama cross-coupling of 1-(triethoxysilyl)buta-1,3-dienes with aryl iodides.

Entry	R (Diene)	Aryl Iodide	Product	Isolated Yield [%]	Selectivity EE/EZ
1	Ph	PhI	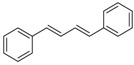	89	99:1
2	Ph	3-NO_2_C_6_H_4_I	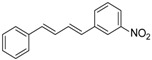	86	>99
3	Ph	4-MeOC_6_H_4_I	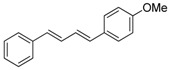	79	>99
4	Ph	2-C_10_H_7_I	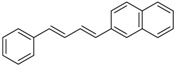	55	99:1
5	MeO	4-MeOC_6_H_4_I	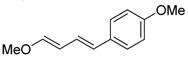	70	95:5
6	MeO	PhI	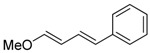	54	98:2
7	Me	4-MeOC_6_H_4_I	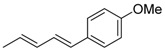	62	99:1

Reaction conditions: [diene]:[aryl iodide]:[TBAF]:[Pd_2_(dba)_3_] =1:1.2:2:0.02; THF, 65 °C, 24 h.

**Scheme 3:**
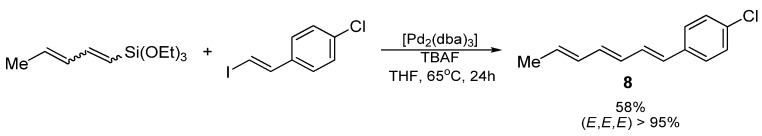
Synthesis of (*E*,*E*,*E*)-1-(4-chlorophenyl)hepta-1,3,5-triene.

A successful, simple, and selective method for the synthesis of unsymmetrical (*E*,*E*)-1,4-disubstituted buta-1,3-dieneshas prompted us to test the selected 1-(triethoxysilyl)penta-1,3-dienein the synthesis of a stereodefined (*E*,*E*,*E*)-1,3,5-hexatriene derivative by its palladium-catalyzed Hiyama cross-coupling with (*E*)-4-chlorostyryl iodide. The optimal conditions established for the reactions of silyl dienes with aryl iodides were applied to the (*E*)-styryl iodide, providing moderate yield (58%) of the desired (*E*,*E*,*E*)-1-(4-chlorophenyl)hepta-1,3,5-triene 8 (Scheme 3). The Hiyama cross-coupling process proceeded in a highly stereoselective manner to yield product containing (*E*,*E*,*E*)-triene as the predominant compound; however, trace amounts of the respective (*E*,*E*,*Z*) and (*E*,*Z*,*Z*) isomers (<5%) were also detected using the GC-MS method. The structure of synthesized triene was confirmed by GC-MS and NMR spectroscopy(see Supporting Information; Figures S15 and S16).

## 3. Experimental Section

### 3.1.General Procedure for the Synthesis of (E,E)-1,4-disubstituted buta-1,3-dienes (1–7)

A mixture composed of 1 mmol of 1-(triethoxysilyl)buta-1,3-diene with THF (10 mL) was placed under Ar atmosphere in a Schlenk bomb flask fitted with a plug valve. At room temperature, 2 mmol of TBAF (1M solution in THF) were added and the mixture was stirred for 10 minutes. After this time, 1.2 mmol of the respective aryl iodide and 0.02 mmol (18.3 mg)of [Pd_2_(dba)_3_] were added and the reaction mixture was stirred under argon for 24 h at 65 °C. After the reaction was completed (GC-MS analysis), the volatiles were evaporated under vacuum and the crude product was chromatographed on silica gel (eluent—hexane/ethyl acetate 8:2) to afford the analytically pure products.

The structures of synthesized (*E*,*E*)-1,4-disubstituted buta-1,3-dienes were confirmed by GC-MS and NMR spectroscopy matching data reported in the literature: (*E*,*E*)-1,4-diphenylbuta-1,3-diene 1 [35], (*E*,*E*)-1-(2-naphthyl)-4-phenylbuta-1,3-diene 4 [38], (*E*,*E*)-1-(4-methoxyphenyl)penta-1,3-diene 8 [39].

#### 3.1.1. (*E*,*E*)-1-methox-4-(4-methoxyphenyl)buta-1,3-diene (5); yellow oil; Yield: 0.13 g (70%)

^1^H NMR (300 MHz, CDCl_3_): δ = 3.86 (s, 6H), 5.78 (d, 1H, *J* = 16.1 Hz), 6.02 (dd, 1H, *J* = 8.8, *J* = 16.1 Hz), 6.34 (d, 1H, *J* = 15.8 Hz), 6.63 (dd, 1H, *J* = 8.8, *J* = 15.8 Hz), 7.02–7.10 (m, 2H), 7.27–7.32 (m, 2H); ^13^C NMR (75 MHz, CDCl_3_): δ = 57.0, 62.0, 110.9, 116.6, 126.4, 128.2, 131.9, 139.5, 153.0, 161.1; MS (EI, 70 eV) m/z (rel. int.): 190.0 (100%), 147.0 (60), 131.0 (25), 115.0 (50), 103.0 (25) 91.0 (50), 77.0 (25), 51 (15); Anal. Calcd for C_12_H_14_O_2_: C, 75.76; H, 7.42. Found: C, 75.66; H, 7.49.

#### 3.1.2. (*E*,*E*)-1-Methoxy-4-(phenyl)Buta-1,3-diene (6); yellow oil; Yield: 0.035 g (54%)

^1^H NMR (300 MHz, CDCl_3_): δ = 3.84 (s, 3H), 6.63 (d, 1H, *J* = 14.8 Hz), 6.88 (d, 1H, *J* = 8.8 Hz), 6.89–6.95 (m, 2H), 7.34–7.39 (m, 3H), 7.47–7.49 (m, 2H); MS (EI, 70 eV) m/z (rel. int.): 160.0 (100%), 145.0 (40), 129.1 (50), 116.0 (90), 103 (50). Anal. calcd for C_11_H_12_O: C, 82.46; H, 7.55. Found: C, 82.65; H, 7.72.

### 3.2. Synthesis of 1-(4-chlorophenyl)hepta-1,3,5-triene (8)

A mixture consisting of 1 mmol of 1-(triethoxysilyl)penta-1,3-diene with THF (10 mL) was placed under Ar atmosphere in a Schlenk bomb flask fitted with a plug valve. At room temperature, 2 mmol of TBAF (1 M solution in THF) was added and the mixture was stirred for 10 minutes. After this time, 1.2 mmol of (*E*)-β-iodo-4-chlorostyrene and 0.02 mmol (0.018 mg) of [(Pd_2_(dba)_3_] were added and the reaction mixture was stirred under argon for 24 h at 65 °C. After the reaction was completed (GCMS analysis) the volatiles were evaporated under vacuum and the crude product was chromatographed on silica gel (eluent—hexane/ethyl acetate 8:2) to afford the analytically pure product.

#### (*E*,*E*,*E*)-1-(4-chlorophenyl)hepta-1,3,5-triene (8); yellow oil; Yield: 0.048 g (58%)

^1^H NMR (300 MHz, CDCl_3_): δ = 1.81–1.83 (m, 3H), 5.77–5.86 (m, 1H), 6.59 (dd, 1H, *J* = 9.2, *J* = 16.3 Hz), 6.65–6.66 (m, 1H), 6.87 (dd, 1H, *J* = 8.5, *J* = 15.9 Hz), 6.95 (d, 1H, *J* = 10.4 Hz), 7.28–7.39 (m, 5H); ^13^C NMR (75 MHz, CDCl_3_): δ = 19.14, 127.56, 128.74, 128.78, 128.87, 129.48, 131.74, 131.95, 133.25, 134.27, 135.71; MS (EI, 70 eV) m/z (rel. int.): 204.0 (80%), 189.0 (100), 169.1 (40), 153.1 (60), 141.0 (50), 124.9 (70). Anal. Calcd for C_13_H_13_Cl: C, 76.28; H, 6.40. Found: C, 76.40; H, 6.52.

## 4. Conclusions

In conclusion, 1-(triethoxysilyl)-substituted buta-1,3-dienes have been applied as new building blocks for palladium-catalyzed Hiyama coupling to yield 1,4-disubstituted (*E*,*E*)-1,3-dienes containing aryl or methoxy groups. Although the starting silyldienes consisted of a mixture of geometrical isomers, the Hiyama coupling proceeded in a highly stereoselective manner to yield products containing (*E*,*E*)-dienes as predominant products. Preliminary results on the application of 1-(triethoxysilyl)-substituted buta-1,3-dienes in the synthesis of (*E*,*E*,*E*)-triene skeleton have also been reported.

## Supplementary Materials

The following are available online at www.mdpi.com/1996-1944/8/11/5378/s1.
