# Supplementary material for: 1-(Triethoxysilyl)buta-1,3-dienes—New Building Blocks for Stereoselective Synthesis of Unsymmetrical (E,E)-1,4-Disubstituted 1,3-dienes
Source: Materials (Basel). 2015 Oct 28;8(11):7250–6. doi: 10.3390/ma8115378 (PMC5458880; doi:10.3390/ma8115378)
Supplement: Supplementary file 1 [file materials-08-05378-s001.pdf]

Supplementary Materials

^{1}H and ^{13}C NMR (or MS) Spectra of Products:

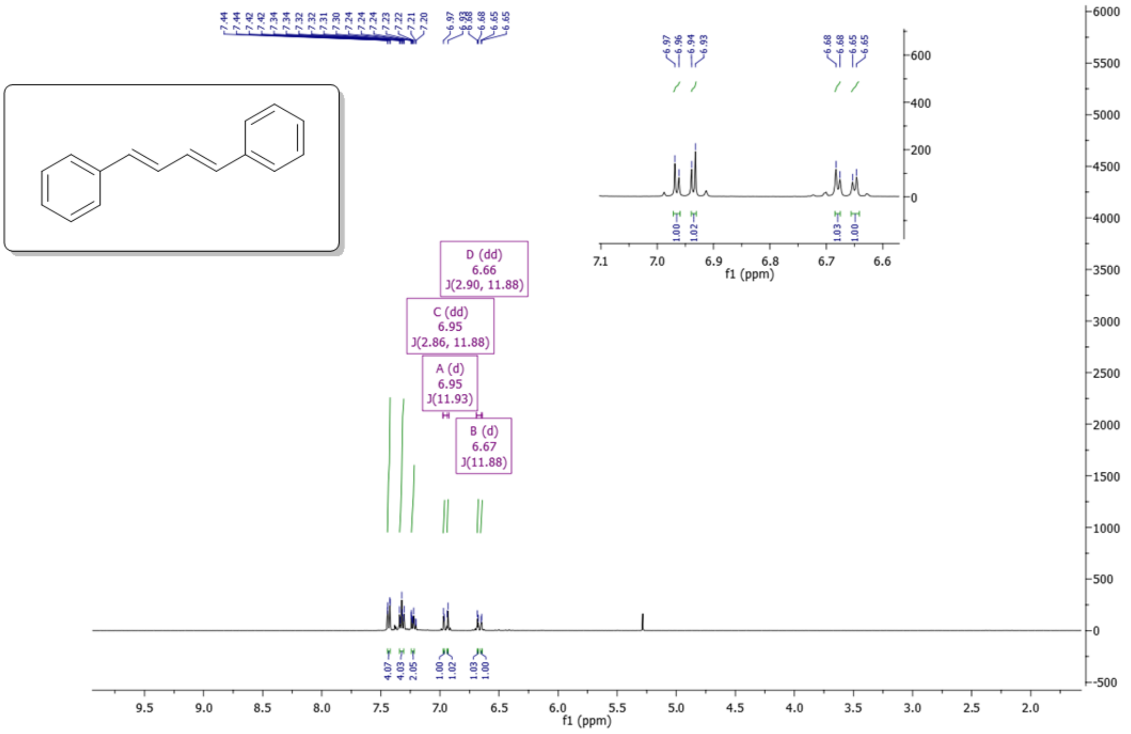

Figure S1. ^{1}H NMR spectrum of (*E*,*E*)-1,4-(diphenyl)buta-1,3-diene (1).

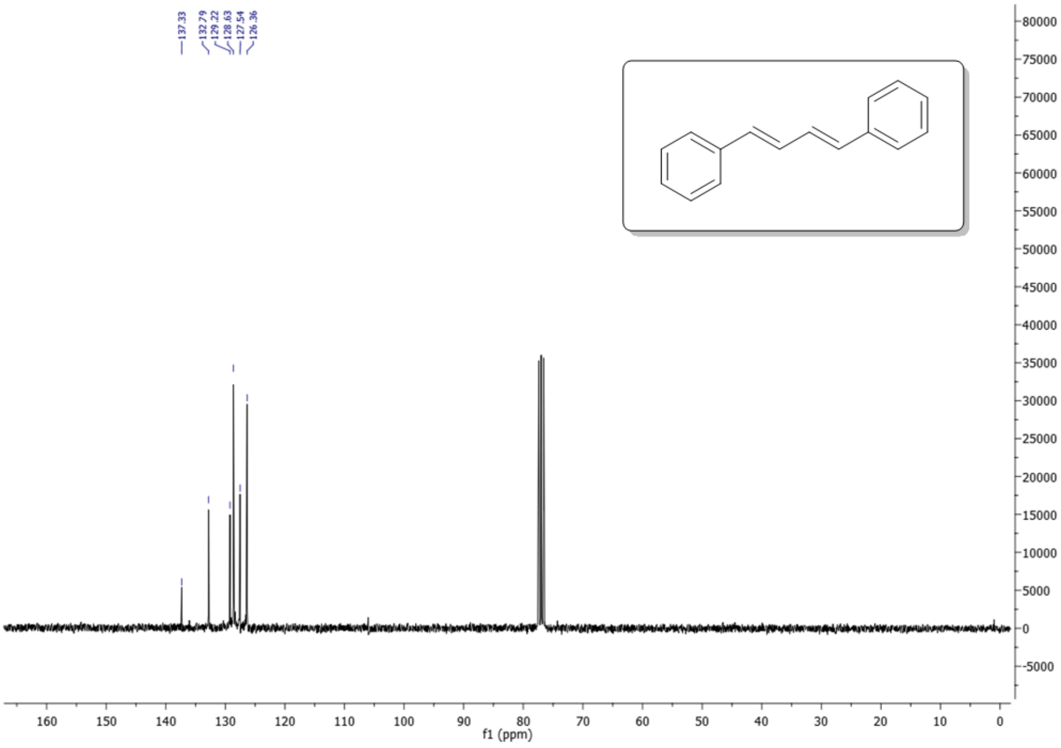

Figure S2. ^{13}C NMR spectrum of (*E*,*E*)-1,4-(diphenyl)buta-1,3-diene (1).

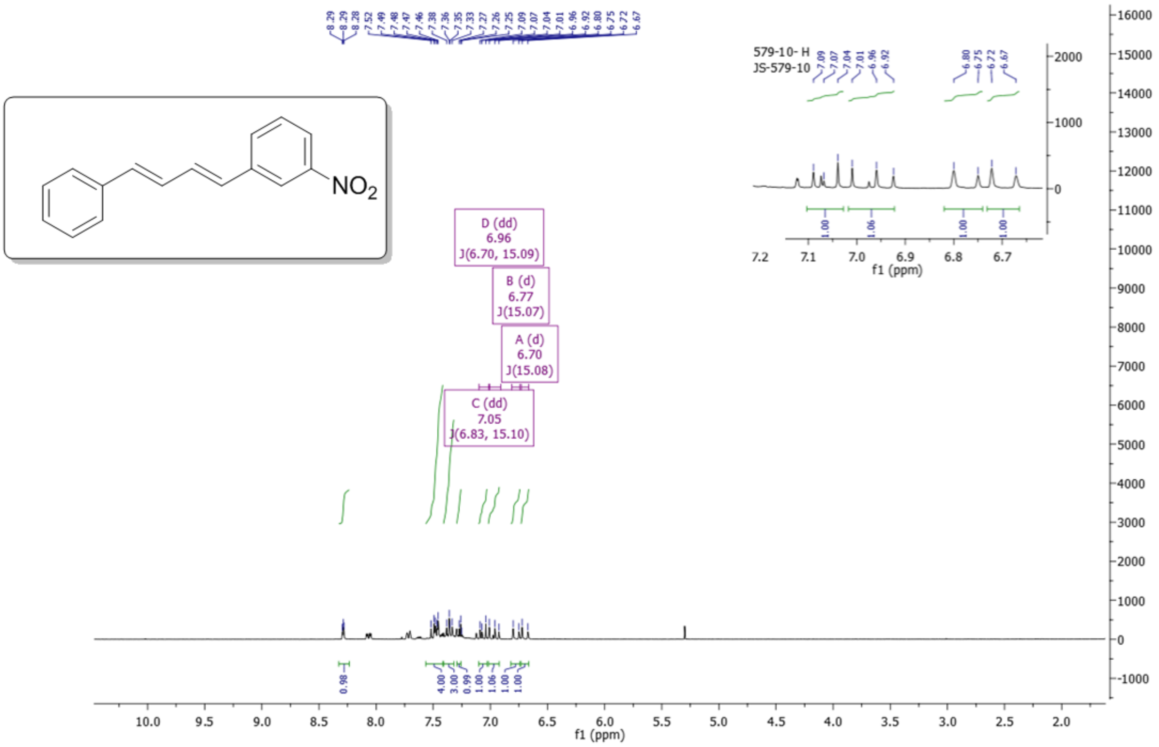

Figure S3. ^{1}H NMR spectrum of (*E*,*E*)-1-phenyl-4-(3-nitrophenyl)buta-1,3-diene (2).

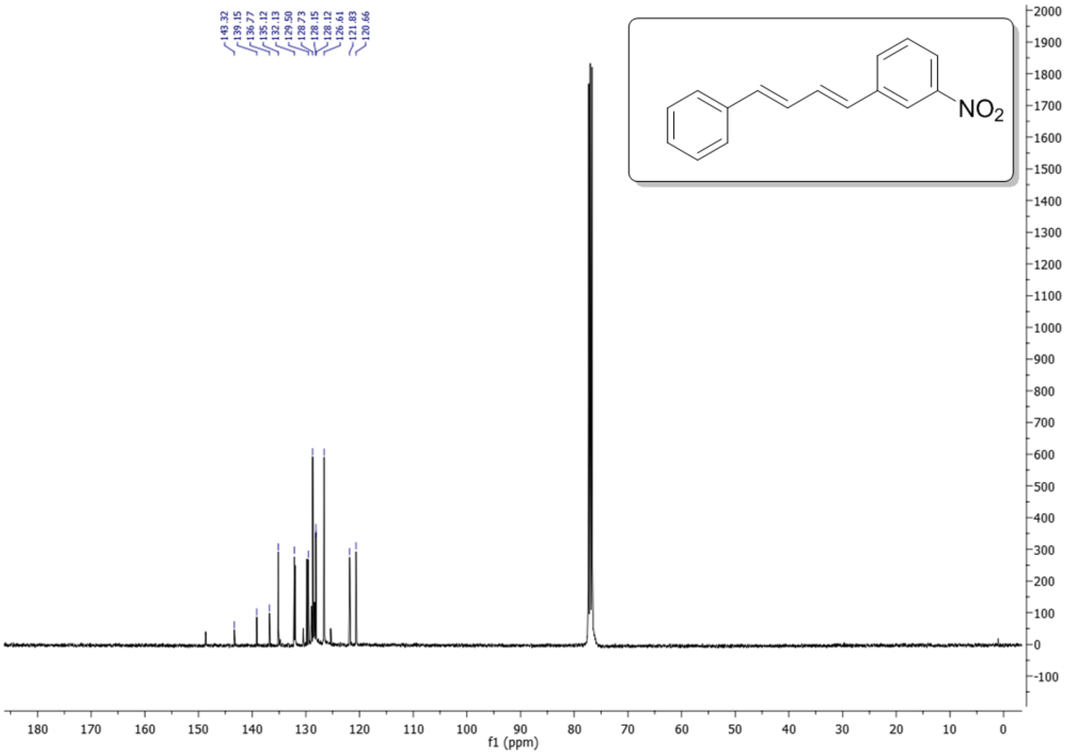

Figure S4. ^{13}C NMR spectrum of (*E*,*E*)-1-phenyl-4-(3-nitrophenyl)buta-1,3-diene (2).

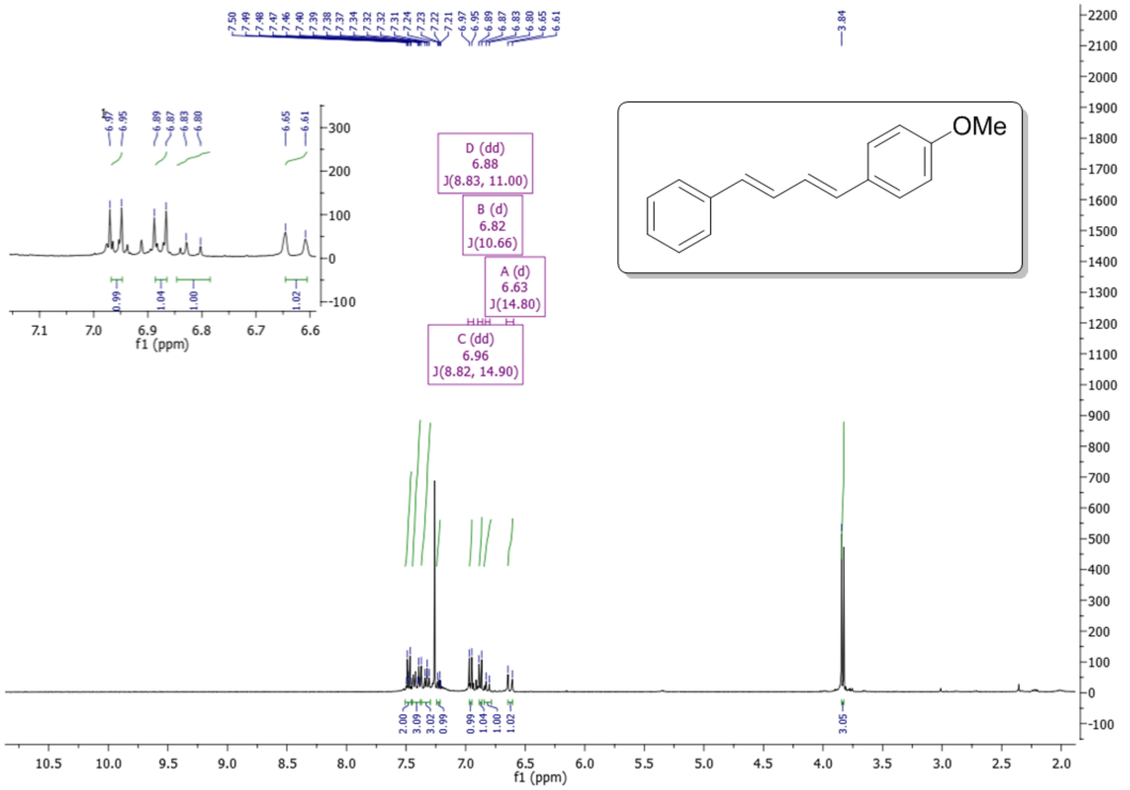

Figure S5. ^{1}H NMR spectrum of (*E*,*E*)-1-phenyl-4-(4-methoxyphenyl)buta-1,3-diene (3).

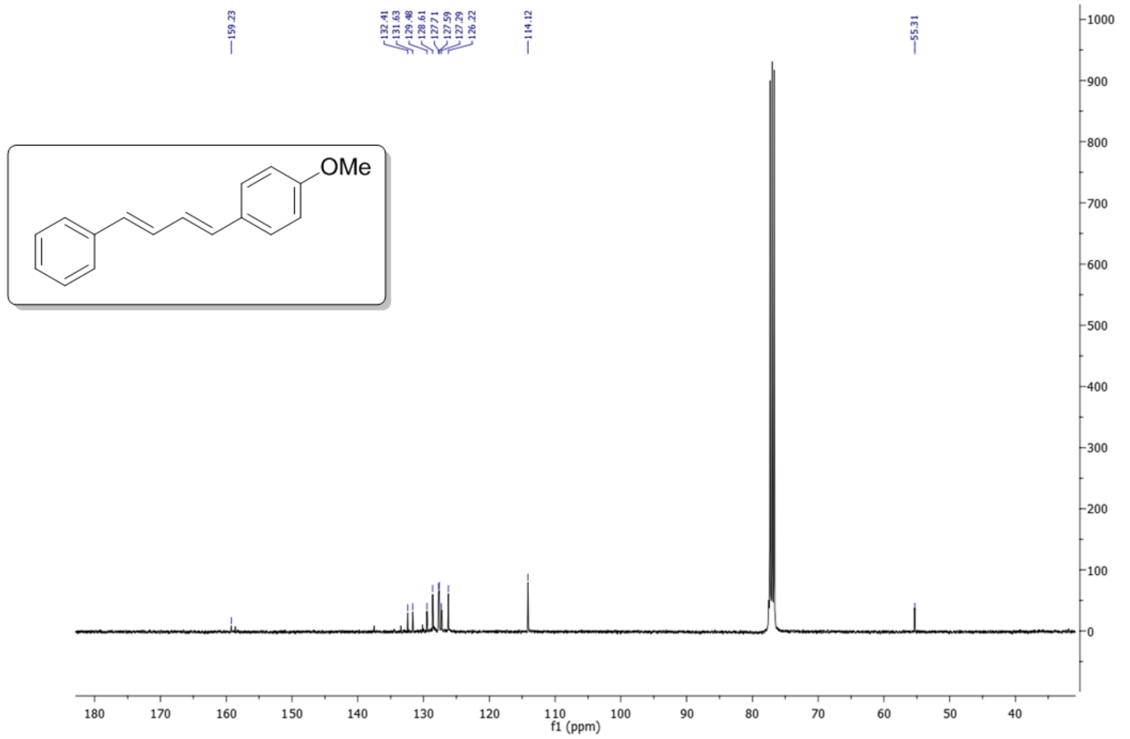

Figure S6. ^{13}C NMR spectrum of (*E*,*E*)-1-phenyl-4-(4-methoxyphenyl)buta-1,3-diene (3).

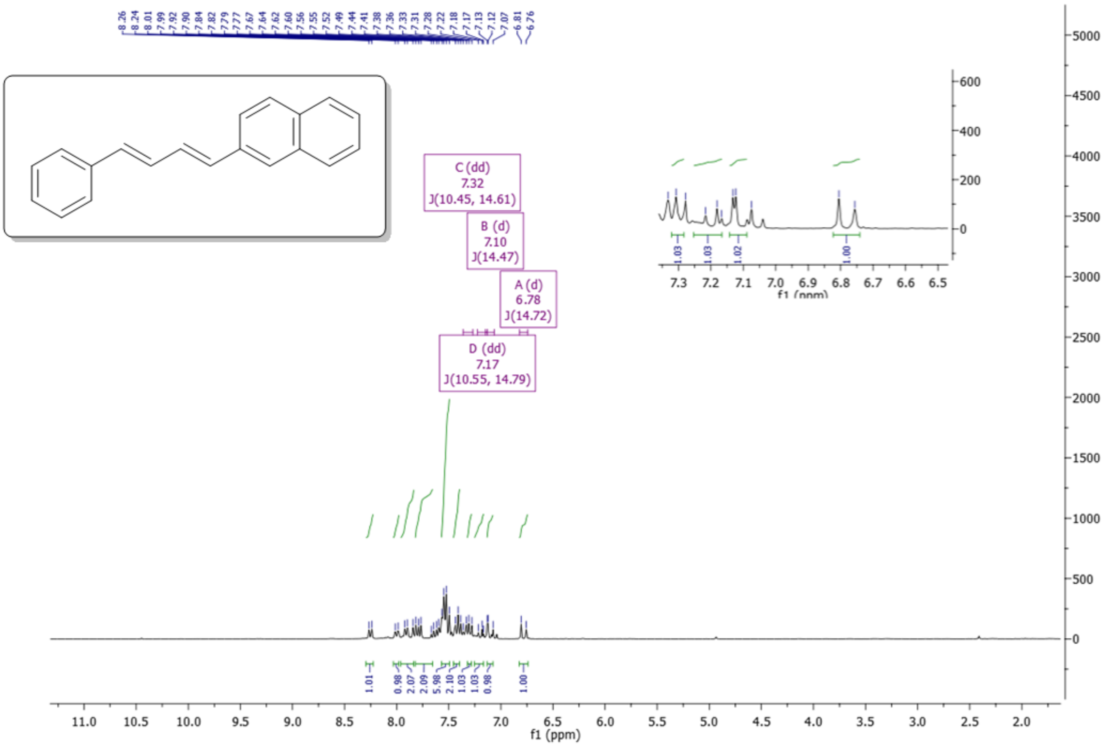

Figure S7. ^{1}H NMR spectrum of (*E*,*E*)-1-(2-naphthyl)-4-phenylbuta-1,3-diene (4).

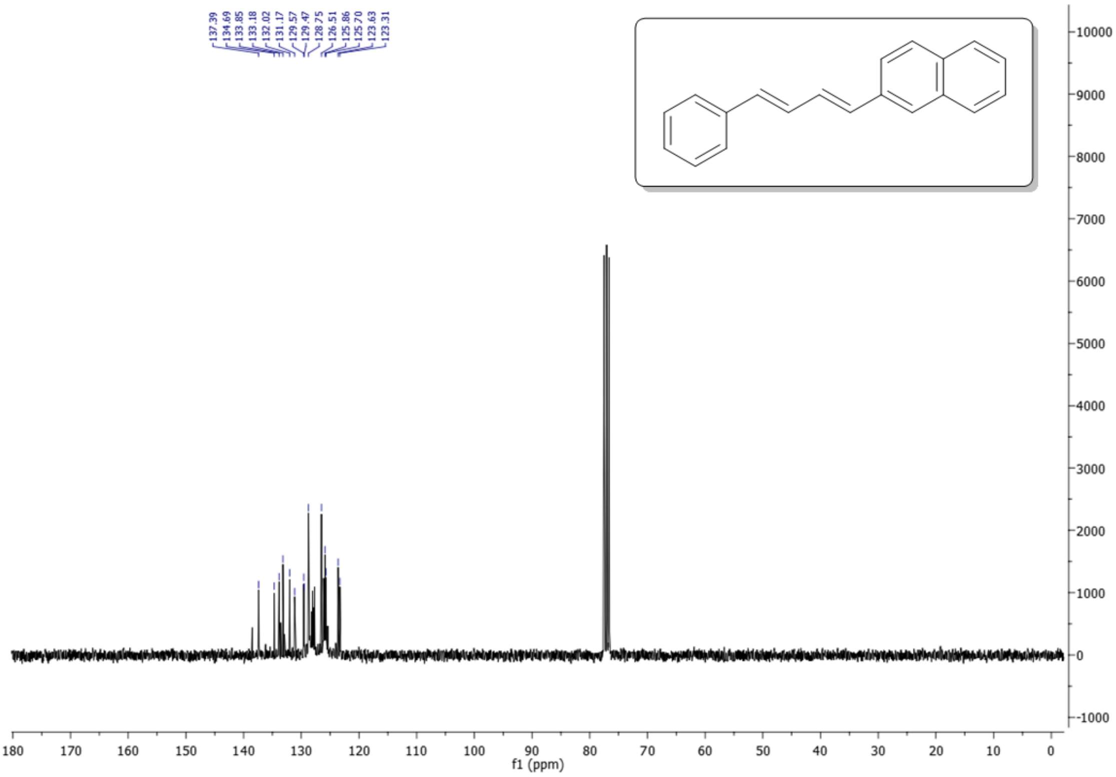

Figure S8. ^{1}H NMR spectrum of (*E*,*E*)-1-(2-naphthyl)-4-phenylbuta-1,3-diene (4).

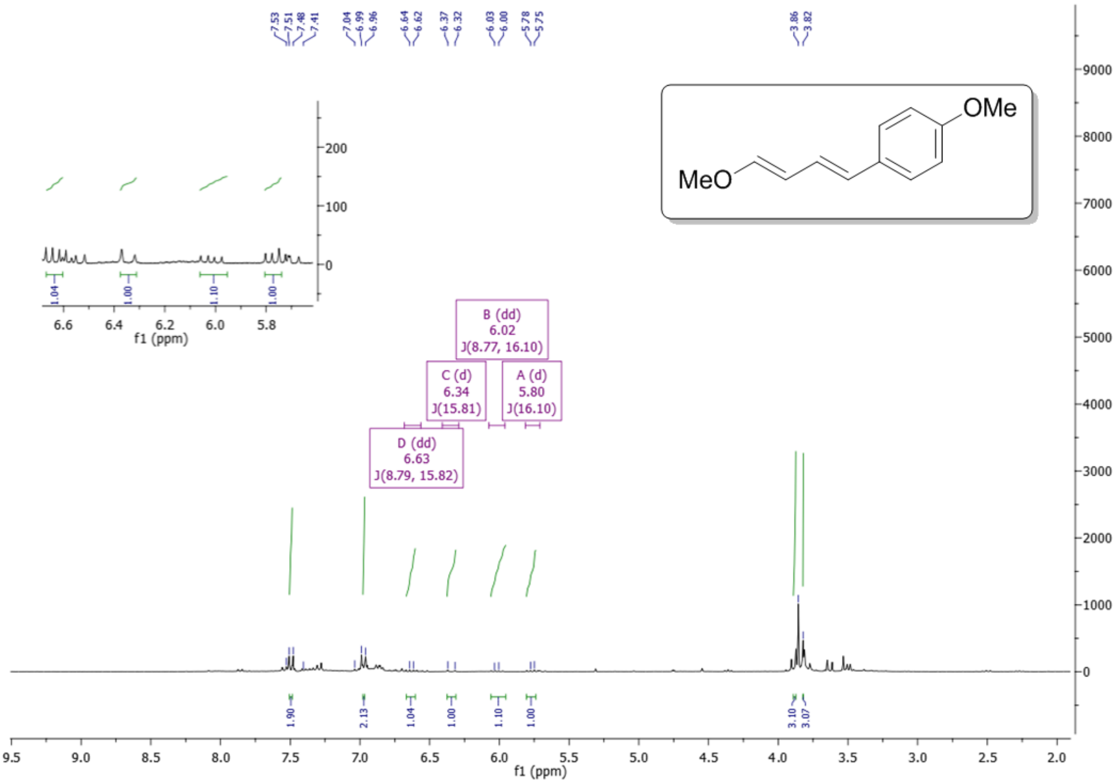

Figure S9. ^{1}H NMR spectrum of *(E,E)-1*-methoxy-4-(4-methoxyphenyl)buta-1,3-diene (5).

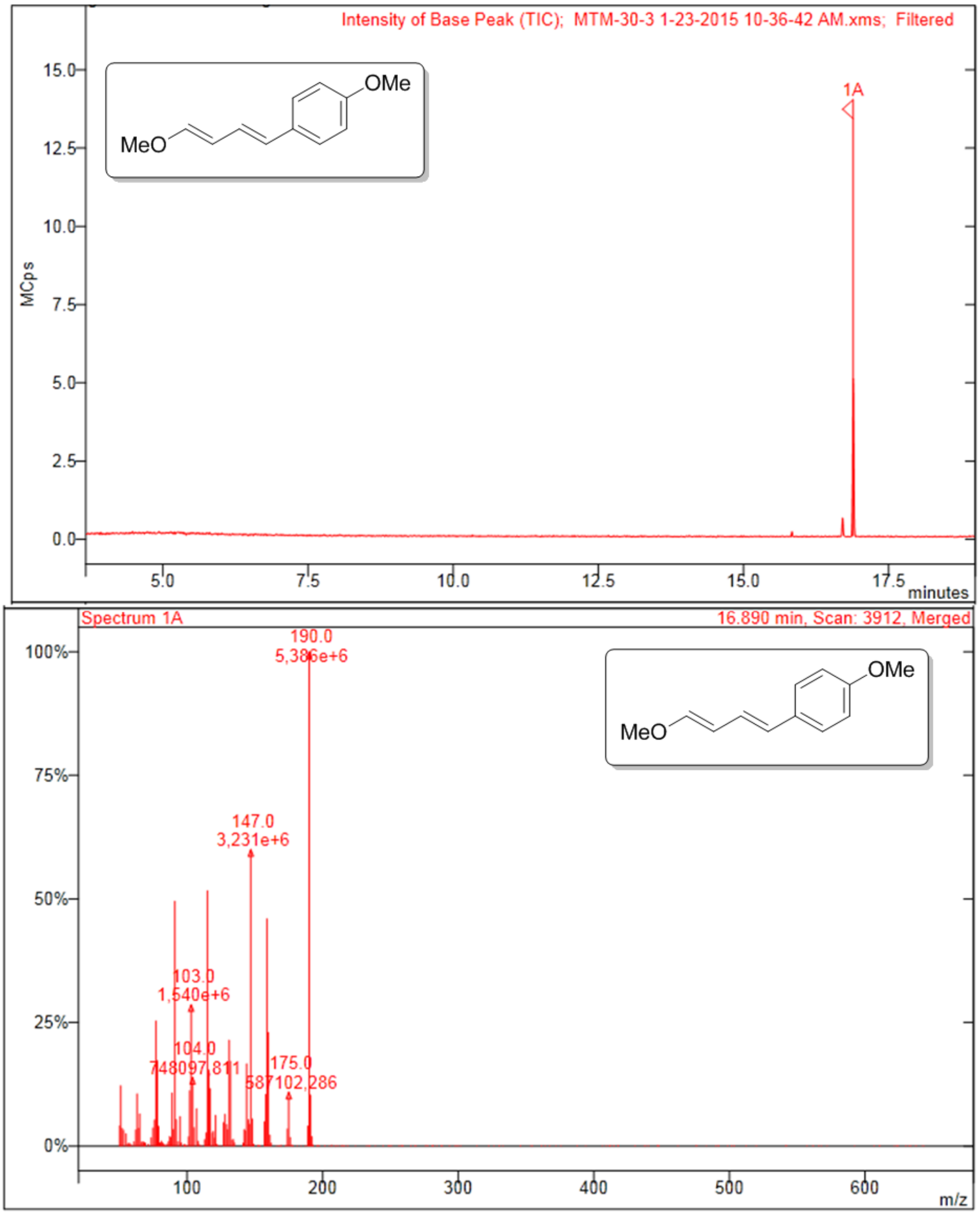

Figure S10. GC-MS spectrum of *(E,E)*-1-methoxy-4-(4-methoxyphenyl)buta-1,3-diene (5).

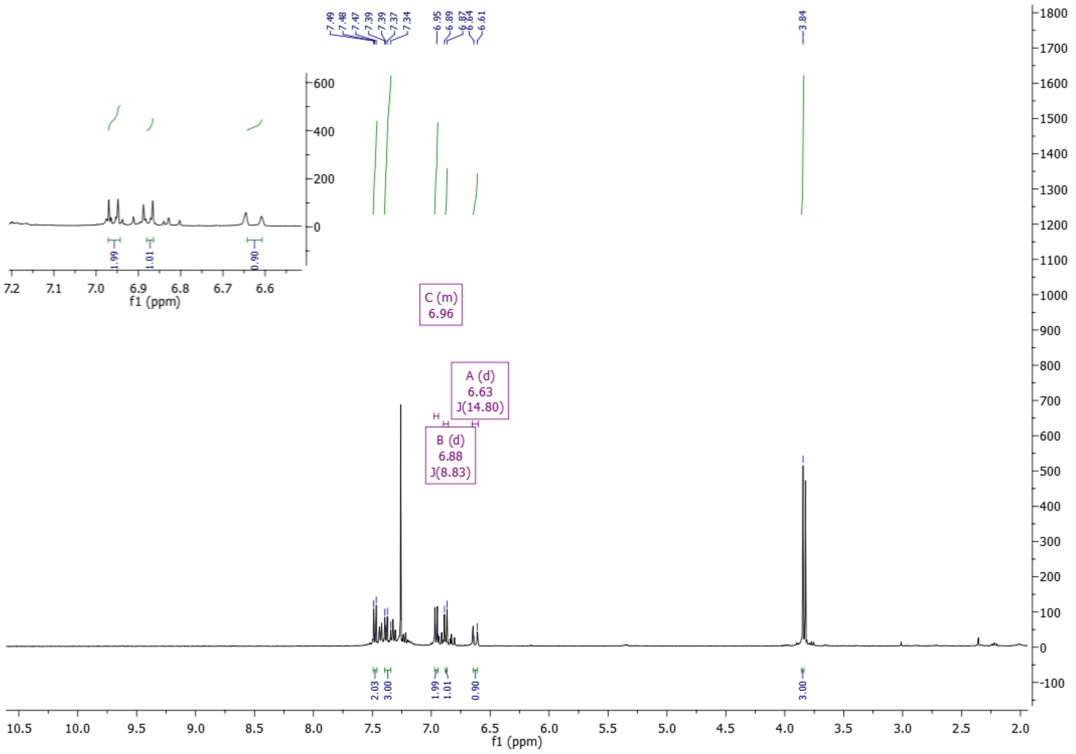

Figure S11. ^{1}H NMR spectrum of (*E*,*E*)-1-methoxy-4-phenylbuta-1,3-diene (6).

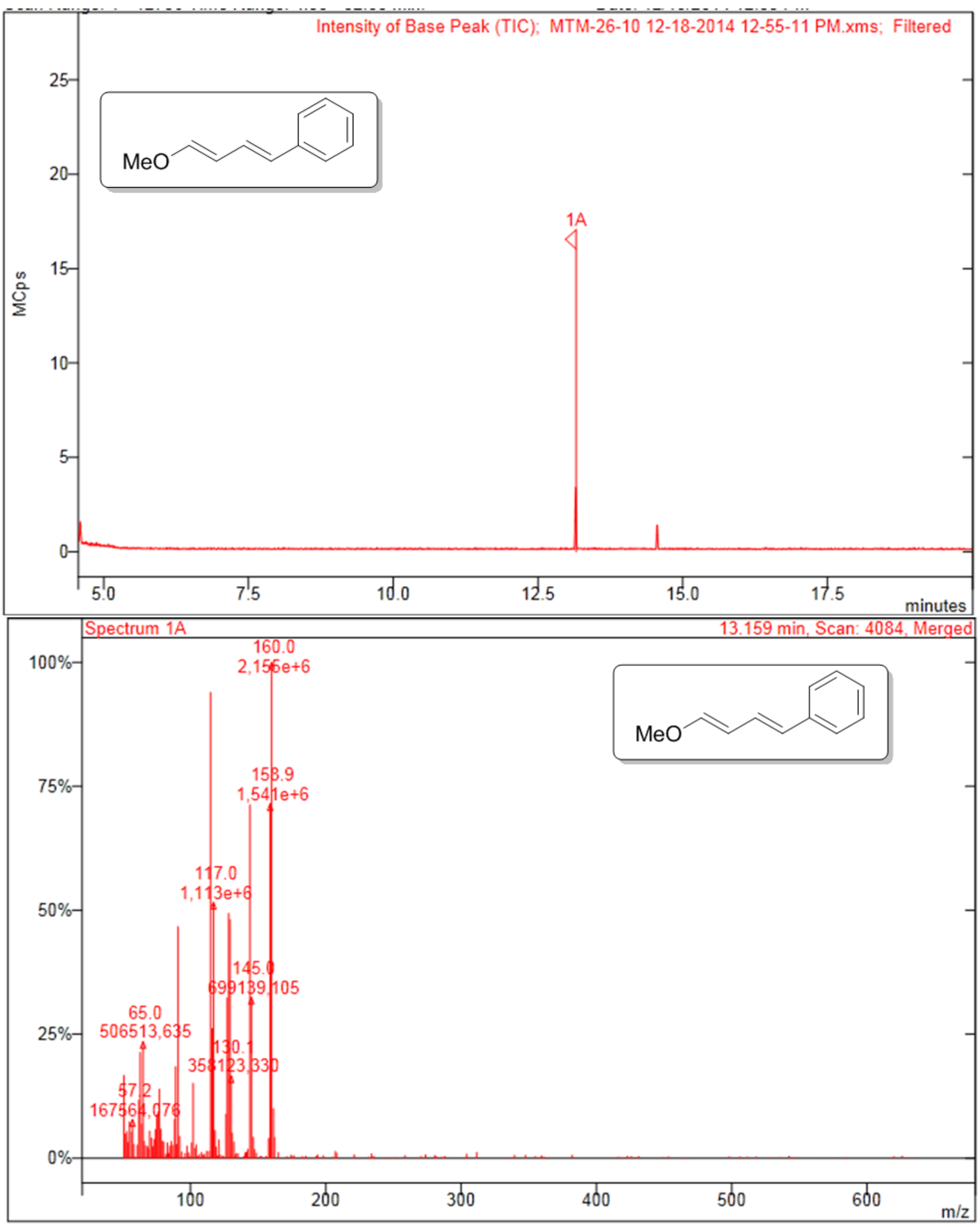

Figure S12. GC-MS spectrum of (*E*,*E*)-1-methoxy-4-phenylbuta-1,3-diene (6).

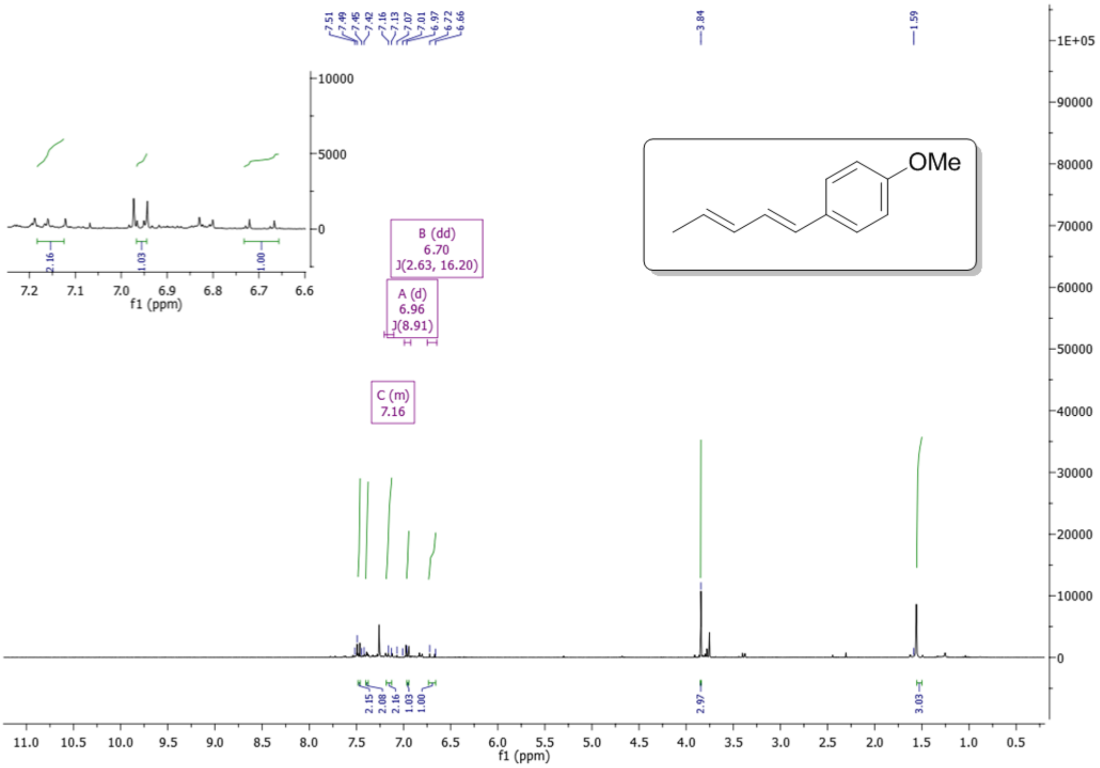

Figure S13. ^{1}H NMR spectrum of (*E*,*E*)-1-(3-nitrophenyl)penta-1,3-diene (*7*).

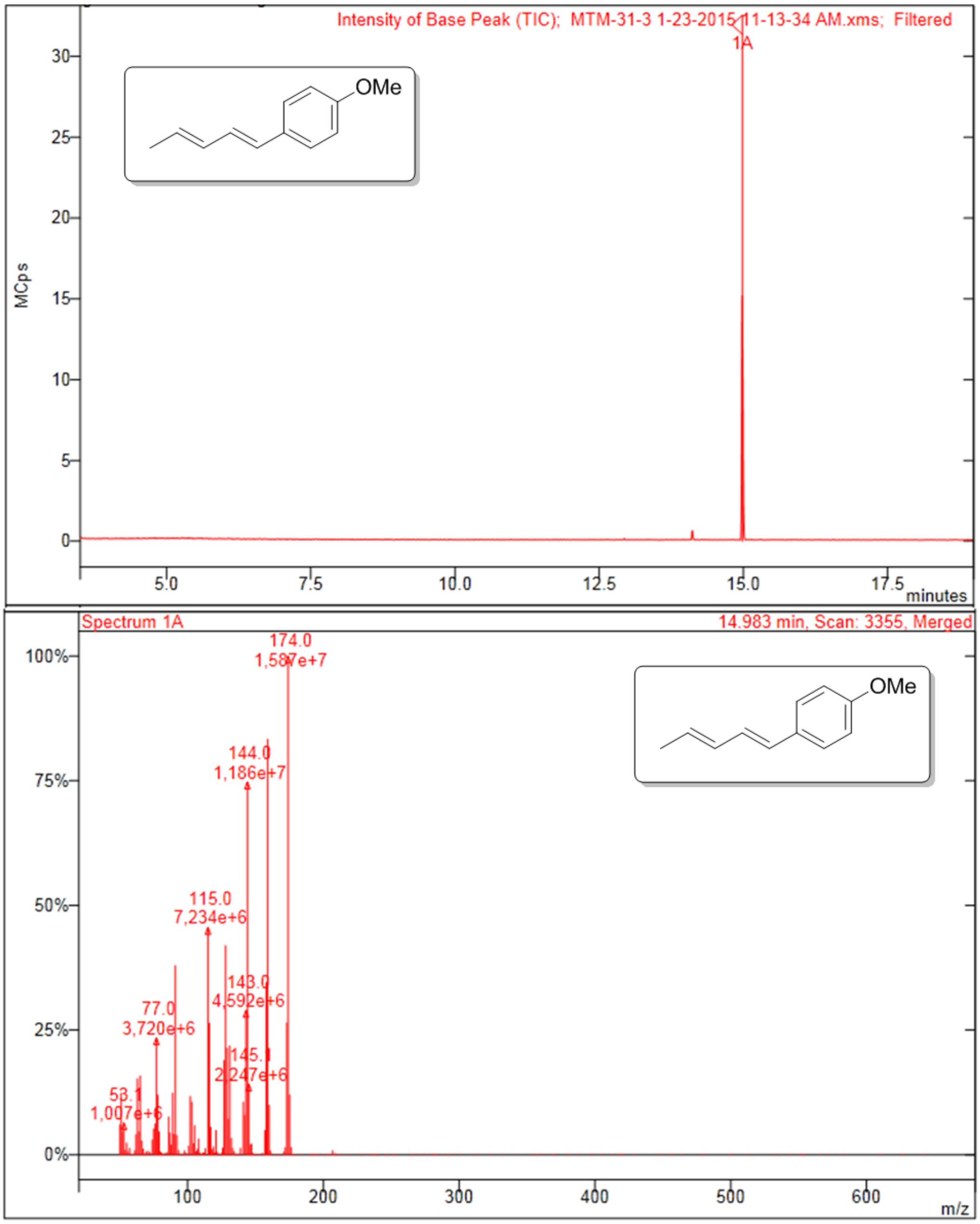

Figure S14. GC-MS spectrum of (*E*,*E*)-1-(3-nitrophenyl)penta-1,3-diene (7).

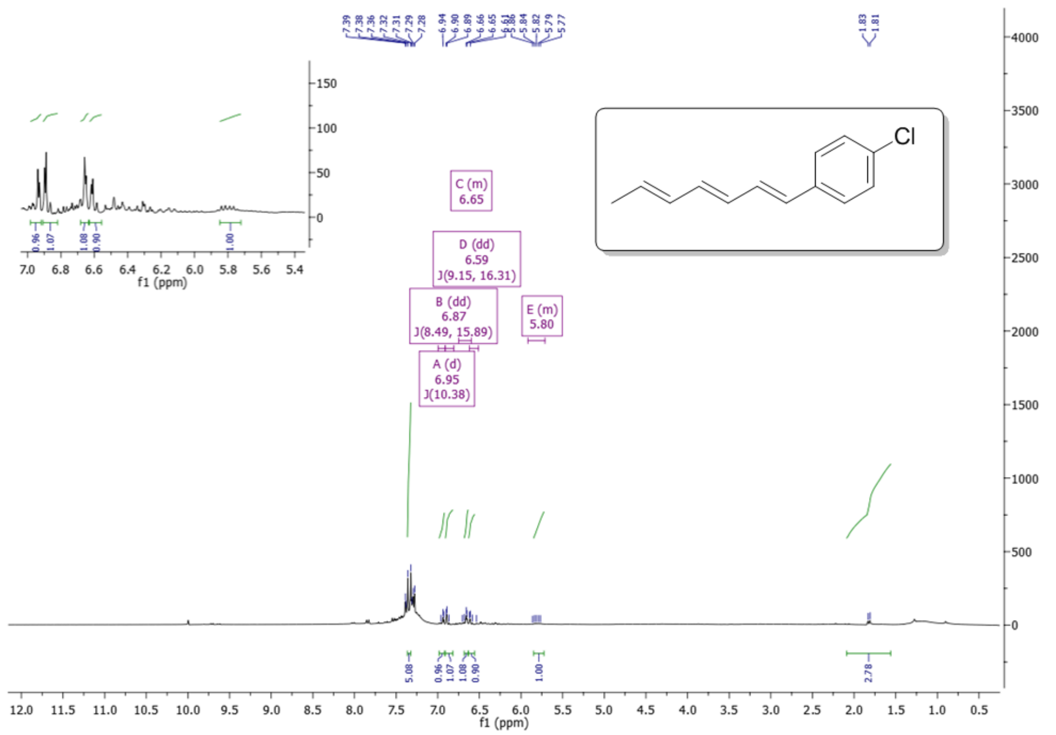

Figure S15. ^{1}H NMR spectrum of (*E*,*E*,*E*)-1-(4-chlorophenyl)hepta-1,3,5-triene (8).

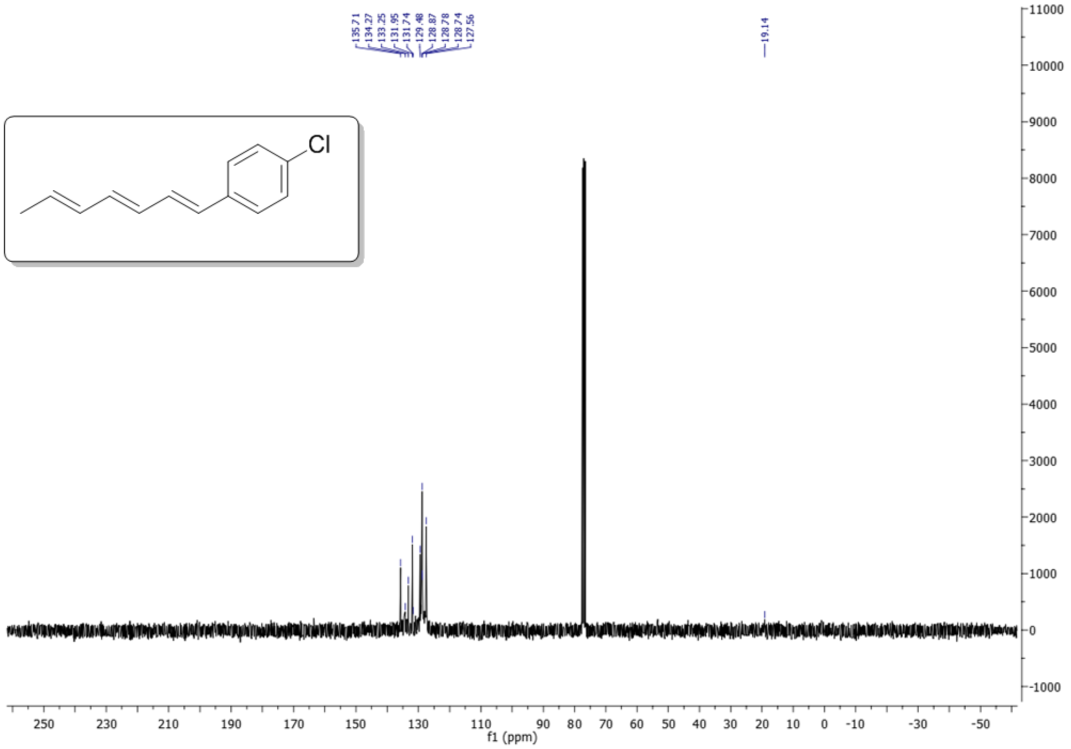

Figure S16. ^{13}C NMR spectrum of (*E*,*E*,*E*)-1-(4-chlorophenyl)hepta-1,3,5-triene (8).
